# HOW OLDER REFUGEES IN THE US COPED DURING COVID-19 PANDEMIC: A QUALITATIVE RESEARCH STUDY

**DOI:** 10.1093/geroni/igae098.2331

**Published:** 2024-12-31

**Authors:** Jonix Owino

**Affiliations:** Sacred Heart University, Stratford, Connecticut, United States

## Abstract

Due to the novelty experience posed by COVID-19, it is important to understand how older refugees coped in their host communities. This is because older refugees have increased vulnerabilities compared to host populations such as limitations related to healthcare accessibility, language barriers, and discrimination, that may have heightened the challenges posed by COVID-19. As such, the present study examined implications of COVID-19 among older refugees and how they coped. The present study recruited thirty-seven Congolese refugees, aged 55-67 (m=65.7 years) from a community in the East Coast region of the US to participate in interviews on their perceptions of life during COVID-19. The interviews lasted between 35-65 minutes. The qualitative interviews were analyzed using thematic content analysis. Emergent themes showed that though older Congolese refugees in the US experienced COVID-19 challenges similar to other populations, these challenges seemed salient for older refugees compared to non-refugee populations due to refugee background and experiences. For instance, older refugees experienced increased social isolation due to limited access to language interpreters. Moreover, older refugees’ coping strategies during COVID-19 e.g., the use of traditional/herbal medicine and cognitive reframing seemed to have been influenced by the interplay of factors related to their unique refugee experiences and their cultural norms and values. Understanding the implications of COVID-19 among older refugees in the US is beneficial to practitioners in determining how they may assist their clientele in coping with specific situations, considering their specific histories and cultures.

